# The effect of psychological factors on financial behaviour among older Australians: Evidence from the early stages of COVID-19 pandemic

**DOI:** 10.1371/journal.pone.0286733

**Published:** 2023-06-08

**Authors:** Vandana Arya, Rajabrata Banerjee, Braam Lowies, Christa Viljoen, Kurt Lushington

**Affiliations:** 1 UniSA Business, University of South Australia, Adelaide, South Australia, Australia; 2 Department of Financial Management, University of Pretoria, Hatfield, Pretoria, South Africa; 3 UniSA Justice and Society, University of South Australia, Adelaide, South Australia, Australia; Al-Zaytoonah University for Science and Technology, STATE OF PALESTINE

## Abstract

The current study investigated the association between psychological factors and financial behaviour during the COVID-19 pandemic in older people. Older people were chosen compared to other age groups because of the relatively greater impact in this age group of suboptimal financial decisions on future financial wellbeing. We hypothesised that the psychological factors facilitating general wellbeing during the COVID-I9 pandemic, i.e., positive mental wellbeing, hope, and positive coping, will have positive effects on financial behaviour. Based on telephone interviews, 1501 older Australians (Men = 750 and Women = 751; 55-64y = 630; > 65y = 871) completed an omnibus questionnaire examining coping, hope, mental wellbeing, and financial behaviour. Data was analysed using logistic regression and an ordinary and two-stage least square frameworks. Analyses revealed that the psychological factors identified as facilitating general wellbeing during the COVID-I9 pandemic also facilitated positive financial behaviour with hope and mental wellbeing emerging as significant determinants. Based on weightings from principal component analysis, one item each from the hope and mental wellbeing scale with eigenvalues > 1 were found to be robust predictors of positive financial behaviours. In conclusion, the findings support the assumption that the psychological factors associated with general wellbeing during the COVID-19 pandemic are also associated with positive financial behaviour. They further raise the possibility that single hope and positive mental well-being items can also be used to monitor psychological health and predict financial behaviour in older people and, in particular, at times of crisis. The latter may be useful measures for government to monitor psychological and financial wellbeing and inform policy for supporting older people at times of crisis.

## Introduction

The COVID-19 pandemic has had a profound impact on markets and accordingly has influenced individual financial behaviours: i.e., the intention to save, controlling spending, paying bills on time, planning for one’s financial future, and providing for oneself and family [1]. A population especially vulnerable to the financial effect of the COVID-19 pandemic are older people [1–4]. In most developed nations, retirement is typically characterised by high asset levels but limited cash reserves and, therefore, a lower resilience to debt [5]. The risk of poor financial wellbeing is highest in older people with limited income, inadequate health care, being reliant on market-linked investments, and those with limited financial knowledge, skills, and motivation [1, 6–8]. In older Australians this susceptibility is further heightened if they are renters (notably older women), unemployed (notably non-English speakers), from an older age group (e.g., > 65 years) and have a disability [3, 9, 10].

The COVID-19 pandemic has also led to high levels of psychological distress [11, 12]. This however has proven responsive to mitigation with studies pointing to the beneficial effects of protective factors such as hope, coping and positive mental wellbeing [2, 13–15]. These factors are also associated with personality traits (i.e., openness, conscientiousness, etc) known to promote psychological health during the COVID-19 pandemic [16–20]. It is likely at a time of crises, such as the COVID-19 pandemic, that these same psychological factors will be associated with positive financial behaviours. Research conducted prior to the COVID-19 pandemic indicates that hope has been associated with proactive financial behaviours and reduced financial risk seeking [21–23], positive coping with lower indebtedness [24, 25], and positive mental wellbeing with positive financial behaviour and wellbeing [26]. More recently, positive mental wellbeing is reported to moderate the impact of economic and psychosocial stressors arising from the COVID-19 pandemic [4, 27].

**Fig 1:**
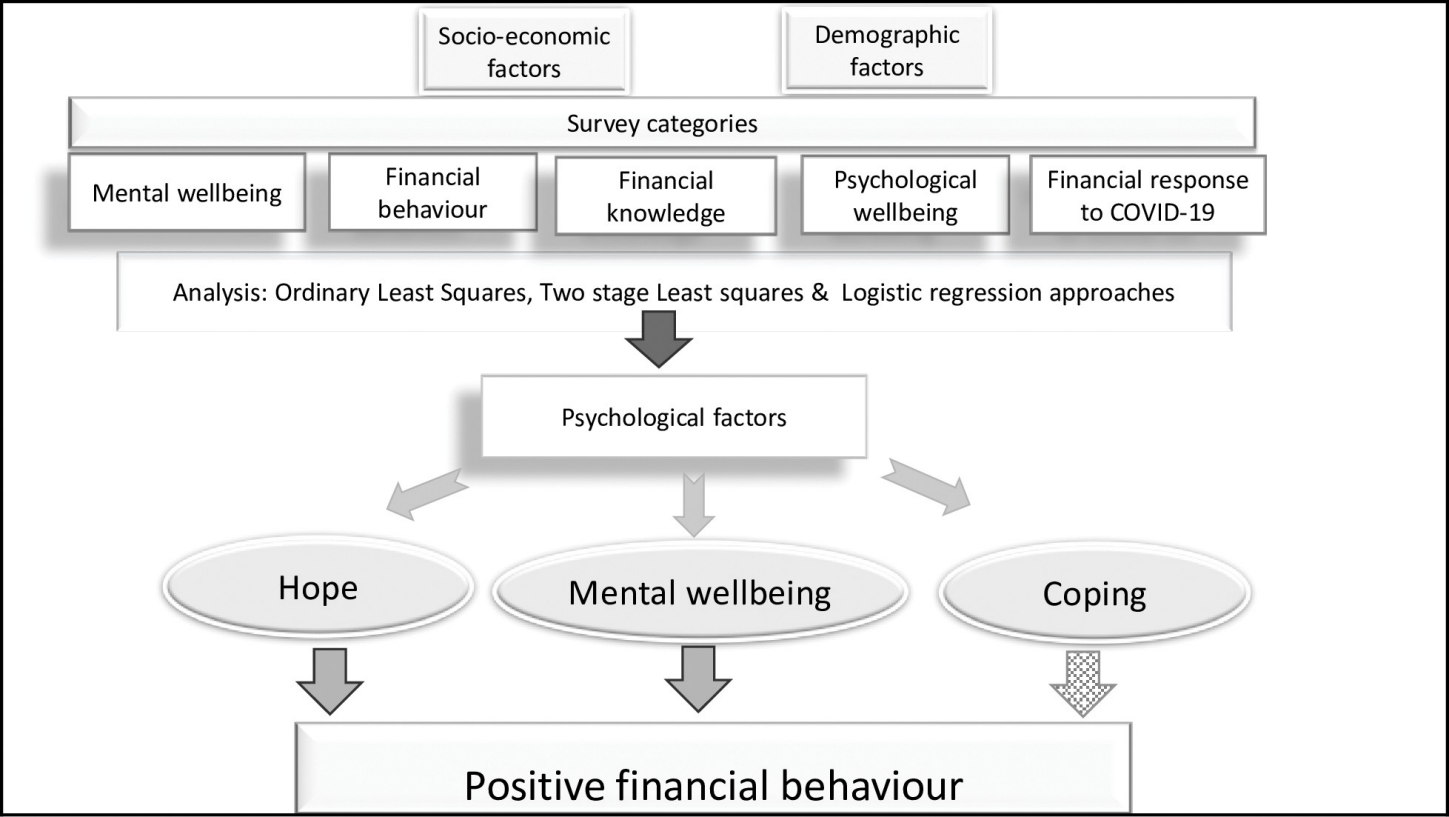
Synopsis of findings, highlighting independent variables, analysis, and impact on dependent variable (positive financial behaviour).

In sum, a better understanding of the association in older Australians between protective psychological factors and financial behaviour at a time of crisis such as the COVID-19 pandemic is timely. The aim of this study is to examine the association between three key protective psychological factors, hope, coping and mental wellbeing on financial behaviour using cross-sectional survey data from a survey of older Australia. It is noted that studies have used cross-sectional data to examine, for example, the impact of the COVID-19 pandemic on psychological health and [28, 29] employment [28–31]. In this study, we further propose not only to examine potential associations but also to infer causality by examining the effects of psychological factors on financial behaviour after controlling for reverse causality using an instrumental variable approach in a two-stage regression framework [32]. It is hypothesized after controlling for known confounds (i.e., gender, employment status, non-English speaking background, homeownership, age, financial socialisation, and disability) that older people during the COVID-19 pandemic with higher hope, coping and mental wellbeing will report a higher frequency of positive financial behaviours. A synopsis of this study, including a visual representation of the findings is presented in Fig 1.

## Materials and methods

### Data, participants, and design

The data used in this study was obtained from the Financial and Psychological Wellbeing Survey (FPWS) which assessed the impact of the COVID-19 pandemic on the psychological wellbeing and financial decision-making of older Australians either planning to, or retired [33]. The report contains full details of the survey methods, questionnaire, and processes. In brief, the FPWS assessed five broad areas: demographics; financial behaviour; financial knowledge; psychological and mental wellbeing; and response to the COVID-19 pandemic. Whilst psychological wellbeing refers to the overall emotional and cognitive state of an individual, including their feelings of happiness, life satisfaction, and a sense of purpose; mental wellbeing is a subset of psychological wellbeing and focuses more on the cognitive and emotional aspects of an individual’s mental health. In this study, mental wellbeing aspects of an individual were chosen to be examined. The data was collected in February 2021 using a simple random sampling method. The sample was stratified to ensure balanced gender and age distribution (young-old (55–64 years) versus old-old (≥ 65y)) [9], with a proportional representation of 900 residents living in every major Australian capital city and 600 residents from regional/rural regions of Australia. Due to higher population concentrations, major cities comprised of 60% of the total sample with inner and outer regional areas representing 20% of the sample each. Screening was conducted by rejecting any potential participant younger than 55 years of age to capture responses of the older cohorts of the population. The data collection process was administered by the Edith Cowan University Survey Research Centre, using computer-aided telephone interview (CATI) technology. After initial screening, 1 501 older Australians completed the survey. The survey was developed by a research team consisting of finance and psychology experts and pilot tested for effectiveness and robustness and modified after consultation with representatives from finance and aged-care industries. Based on individual postcodes, the Australian Bureau of Statistics Index of Relative Socio-economic Advantages and Disadvantage scores were used to assess socio-economic status (SES) (1 = lowest to 10 = highest SES) [34]. These were then collapsed into three bands low (decile 1–3), mid (decile 4–7) and high (decile 8–10) SES. The study was approved by the Human Research Ethics Committee of University of South Australia (Ethics approval: 86/2020).

### Measures

#### Hope and general wellbeing

Following the literature, the Adult Hope Scale was used to assess hope [35]. Participants were asked ‘How well the following statements apply to you in general’ and to rate themselves using a five-point scale (1 = *Very well* to 5 = *Does not apply at all*). The Adult Hope Scale generates two scales containing four items each: Agency (goal-directed energy (e.g., *I energetically pursue my goals*)) and Pathways (planning to accomplish goals (e.g., *Even when others are discouraged*, *I know I can find a way to solve the problem*). It also contained four filler items (*I feel tired most of the time*, *I am easily downed in an argument*, *I worry about my health*, and *I usually find myself worrying about something*). The Adult Hope scale is widely used and reported to have good reliability and validity [36].

#### Coping

Coping was assessed using Stallman’s newly developed twenty item Coping Index [37]. Respondents were asked ‘How often you do the following things when you are feeling anxious, stressed or distressed’ and items were rated using a four-point scale (0 = *Not at all* to 3 = *Most of the time*). The Coping Index generates two subscales: Positive (e.g., *Take a few deep breaths to calm down*) and Negative (e.g., *Think to yourself in critical*, *harsh*, *or negative way*) coping. The Coping Index is reported to have good predictive validity [38].

#### Mental wellbeing

The WHO-5 Well-Being Index which is a well-validated and widely used instrument was used to assess current mental wellbeing, i.e. how well a person can cope with the normal stresses of life and how they feel about themselves [39]. The WHO-5 contains five items (e.g., *I have felt cheerful and in good spirits*) and respondents were asked to rate ‘How they have been feeling over the last two weeks’ on a five-point scale (1 = *Some of the time* to 5 = *All the time*).

#### Financial behaviour

Financial behaviour was assessed using the six items formulated by Kempson et al. [40, 41]. Respondents were asked ‘Could you please indicate how well the following items apply to you personally’ (e.g., *I am very thorough in my approach to financial planning*) and rate themselves using a five-point scale (1 = *Applies very well* to 5 = *Does not apply very well*). The items have been extensively trialled by the World Bank as part of program to assess financial capability in low- and middle-income countries [41].

Prior to the empirical analyses, the hope, coping, and financial behaviour items were reversed scored so that higher scores indicated higher functioning.

### Statistical analyses

#### Principal component analysis

To construct the indices assessing hope, positive coping, mental wellbeing and financial behaviour, relevant questionnaire items were entered into a series of Principal Component Analyses (PCAs). PCA is an empirical technique that assigns value to each item within a group of items signifying the total amount of variance explained by an item [42, 43]. Items with the variance equal or greater than one indicates that the extracted item explains a significant amount of the variance. Based on the first principal component, only items with an eigenvalue greater >1 was retained which were then aggregated and normalised (values ranged between 0 and 1) (see, the selected items and corresponding Eigen values in Table 1). The STATA statistical package was used to conduct all empirical analyses.

**Table 1:** Principal component analysis.

Variables	Item	Eigenvalue
Financial behaviour—S1	I am very thorough in my approach to financial planning	1.09111
Financial behaviour—S2	I always pay my credit card off each month	1.11743
Mental Wellbeing	I have felt calm and relaxed.	3.05691
Hope	Even when others are discouraged, I know I can find a way to solve the problem.	3.78645
Coping	Think to yourself in critical, harsh, or negative way	4.35339
Financial Wellbeing	I am satisfied with my financial situation	1.67115

#### OLS and logistic regression models

The aggregate indices were then used to examine the relationship between psychological factors and financial behaviour using an ordinary least squares (OLS) framework. The empirical equation used in the formulation was:

$$FB_{i}=\gamma_{0}+\gamma_{1}MW_{i}+\gamma_{2}H_{i}+\gamma_{3}C_{i}+X_{i}+\varepsilon_{i}$$ (1)

where for individual *i*, *FB_i_* denotes financial behaviour, *MW_i_* mental wellbeing, *H_i_* hope, and *C_i_* coping. *X_i_* refers to an array of controls to capture individual characteristics and *ε_i_* the idiosyncratic error term. To understand the associations between psychological factors and financial behaviour and to avoid multicollinearity, the indices were each considered individually in separate empirical specifications. As the following are known to influence individual financial behaviour they were controlled for in the analyses: gender (Men, Women), employment status (Employed, Unemployed), culture (English speaking at home, Non-English speaking at home), accommodation type (Outright homeowner, Renting/Mortgaged), financial socialisation (Individual, Joint financial decision-making behaviour), disability (No, Yes), age group (Young-old 55–64, and Old-old ≥ 65y), and income level. The control variables were also normalised to generate values between 0 to 1 except income which was categorised into eight separate groups where group 1 indicated a range between $0 and $9,999 and group 8 indicated a value greater than $150,000.

As participants were categorized in two groups (psychological item ‘applies very well or well’ or ‘does not apply very well or well’), binary logistic regression was used for analyses. This approach is appropriate for analysing categorical variables and has been used by previous authors to assess the impact of the COVID-19 pandemic on psychological wellbeing [30, 31].

Robustness was assessed by replacing positive mental wellbeing with a financial wellbeing index. The latter index was generated using the same approach described above and involved undertaking a PCA of the 14-item Financial Wellbeing Scale contained in the FPWS [33] (see Table 1). Participants were divided into two groups, FB∈(0,1) with FB = 1 indicating a particular financial behaviour item applied ‘*very well* or *well’* and FB = 0 ‘*doesn’t apply very well*, *well or neither well*, or *badly’*. The probability of good financial behaviour was defined as Pr(FB = 1) = p, and not having good financial behaviour as Pr(FB = 0) = 1-p. The same approach was used to categorise participants according to hope, coping, mental wellbeing, and financial wellbeing responses. The empirical specification for the binary outcome based on the odds ratio was:

$$P_{i}=\gamma_{0}+\gamma_{1}MW_{i}+\gamma_{2}H_{i}+\gamma_{3}C_{i}+X_{i}+\varepsilon_{i}$$ (2)

where P = p/(1-p) indicated the odds ratio. MW, H and C were binary variables with values of one when the item response was ‘*very well or well’*, and zero otherwise. X reflected a vector of other controls as defined in Eq (1).

For ease of interpretation, the marginal effects of the discrete explanatory variables on the conditional probability of FB = 1 was calculated. The marginal effect was defined as a change in the likelihood of increase in financial behaviour, Pr(FB = 1), when mental wellbeing, MW∈(0,1), changed from zero to one keeping other variables constant. The marginal values for MW were calculated as follows:

Marginalvalue=Pr(FB=1|MW=1,Z)−Pr(FB=1|MW=0,Z) (3)

where Z was the vector of all other variables in Eq (2) that were considered constant while MW changed from zero to one. Thus, when interpreting results in Eq (3), the estimates provided the changes in FB due to marginal change in MW. Similar interpretations can be made for hope, coping, and financial wellbeing.

Finally, to establish causality between psychological factors and financial behaviours, an instrumental variable approach was adopted in a cross-sectional setting [32]. The estimates from Eq (2) can suffer from endogeneity concerns of simultaneity and reverse causality biases. A reverse causality bias would be considered evident when financial behaviour was found to influence participant’s hope, coping, and mental wellbeing (e.g., if more frequent positive financial behaviour results in greater financial wellbeing, then an individual may become more hopeful and better able to cope with financial distress). A simultaneity bias would be also evident when both the financial behaviour and psychological profile of an individual were jointly determined by their income, employment, and the error term in Eq (2). Thus, to control for endogeneity biases, an instrumental variable approach was adopted using a two-stage least squares estimation technique (TSLS). Instrumental variable is a technique that uses a third (exogenous) variable to control for the effects of confounding (endogenous) factors and estimate the casual effect of one variable on another. The third (exogenous) variable is called an instrument and it must be chosen such that it is correlated with the second (endogenous) variable but not directly correlated with the outcome variable. In brief, if there are three variables X, Y and Z, where Y is the dependent variable, X is the independent variable and Z is the third variable; Z would be correlated with X in the equation but not directly correlated with Y. Thus, Z is expected to influence Y through the variable X. Albeit the difficulty in establishing causality in a cross-sectional study, this technique helps to increase the confidence in causal inferences.

Items from the survey were identified as instruments which were related to an individual’s psychological profile but not to their financial behaviour. The items selected from the hope scale was *I feel tired most of the time*; coping *I Pray*; and mental wellbeing *I have woken up feeling fresh and rested*. Checks were also done to test whether these instruments satisfy relevancy and exogeneity conditions of a valid instrument. Specifically, the Cragg-Donald Wald (CDW) F-statistic provided by Stock & Yogo [44] was used to test if the independent variable was exogenous in the presence of multiple endogenous regressors [45].

## Results

### Summary statistics

Summary statistics for the financial behaviour, mental wellbeing, hope, coping items, and other demographic variables are presented in Table 2.

**Table 2:** Descriptive statistics of the survey data.

Variables	Observations	Frequency	Total Observations
*Financial behaviour–S1*: *I am very thorough in my approach to financial planning*			
Apply (= 0)	1061	70.69	
Does Not Apply (= 1)	414	19.33	1501
*Financial behaviour–S2*: *I always pay my credit card off each month*			
Apply (= 0)	1087	71.41	
Does Not Apply (= 1)	414	17.59	1501
*Mental Wellbeing*: *I have felt cheerful and in good spirits*			
More than half of the time (= 0)	1184	85.55	
Less than half of the time (= 1)	117	14.45	1501
*Hope*: *Even when others are discouraged*, *I know I can find a way to solve the problem*			
Apply (= 0)	1117	74.41	
Does Not Apply (= 1)	384	15.59	1501
*Coping*: *Think to yourself in critical*, *harsh*, *or negative way*			
More than half of the time (= 0)	817	54.44	
Less than half of the time (= 1)	684	45.56	1501
*Gender*			
Women (= 0)	751	50.03	
Men (= 1)	750	49.97	1501
*Employment Status*			
Unemployed (= 0)	741	49.36	
Employed (= 1)	760	50.64	1501
*Culture*			
Speak English (= 0)	1418	95.13	
Do not speak English (= 1)	11	4.87	1501
*Accommodation Status*			
Rent/Mortgage (= 0)	471	31.45	
Outright Homeowner (= 1)	1019	68.55	1501
*Decision making*			
Joint (= 0)	859	57.36	
Alone (= 1)	641	41.7	1501
*Disability*			
Disable (= 0)	441	19.38	
Not Disable (= 1)	1059	70.55	1501
*Age Group*			
65+ (= 0)	871	58.03	
55–64 (= 1)	630	41.97	1501
*Income*	1501	100	1501

Table 3 summarises the descriptive statistics of the PCA based aggregated indices of financial behaviour, hope, coping, mental wellbeing, financial wellbeing, and other control variables. Overall, the descriptive statistics presented in Tables 2 and 3 indicated that the variables were within a reasonable range and encompassed enough variations.

**Table 3:** Descriptive statistics of the dependent, independent and control indices.

Variables	N	Mean	Median	Std. dev.	Min	Max
Financial behaviour*	1,501	0.706	0.714	0.101	0	1
Mental Wellbeing*	1,501	0.711	0.750	0.195	0	1
Hope*	1,501	0.668	0.675	0.153	0	1
Coping*	1,501	0.718	0.719	0.110	0	1
Income	1,501	5.350	6	1.023	1	8
Financial Wellbeing*	1501	0.701	0.706	0.198	0	1

Note

*Variables were normalised and converted into aggregate index using principal component analysis. **Variables were constructed as binary and thus statistical values were unavailable.

Income was a continuous variable and was defined in 8 groups for the empirical analysis: (1) $0, (2) $1 to $9 999, (3) $10 000 to $24 999, (4) $25 000 to 49 999, (5) $50 000 to 74 999, (6) $75 000 to 99999, (7) $100 000 to 149 999, (8) ≥ $150 000. A mean and median value of 5.3 and 6, respectively, for income indicated the mean and median group levels as compared to all 8 groups.

### Baseline results

Following Eq (1), the relationship between hope, coping, mental wellbeing, and financial behaviour was examined using an OLS framework. These results are presented in Table 4. Notably, all psychological indices were positively associated with good financial behaviour.

**Table 4:** OLS estimation results with the combined financial behaviour score as the dependent variable.

Variables	(1)	(2)	(3)	(4)
**Mental Wellbeing** †	0.191***			
	(0.017)			
**Hope** †		0.336***		
		(0.033)		
**Coping** †			0.135***	
			(0.045)	
**Financial Wellbeing** †				0.081**
				(0.031)
**Gender**	0.019*	-0.008	-0.013	0.059*
	(0.010)	(0.010)	(0.010)	(0.013)
**Employment status**	0.019**	0.036***	0.015*	0.149***
	(0.011)	(0.011)	(0.011)	(0.061)
**Culture**	0.011	0.008	0.019	0.154
	(0.043)	(0.041)	(0.043)	(0.134)
**Accommodation**	-0.073***	-0.071***	-0.081***	0.033
	(0.011)	(0.011)	(0.011)	(0.011)
**Decision Making**	0.018**	0.031**	0.030**	0.137**
	(0.010)	(0.010)	(0.010)	(0.081)
**Disability**	-0.031**	-0.031**	-0.049**	-0.136**
	(0.011)	(0.011)	(0.011)	(0.071)
**Age group**	0.000	0.004	0.007	0.114*
	(0.011)	(0.011)	(0.011)	(0.111)
**Income**	0.006***	0.006***	0.007***	0.164***
	(0.001)	(0.001)	(0.001)	(0.045)
**Constant**	0.543	0.473	0.590	0.035
	(0.551)	(0.450)	(0.457)	(0.018)
**R** ^**2**^	0.406	0.438	0.381	0.417
**N**	1501	1501	1501	1501

Note

†Variables were converted into index using principal component analysis (PCA).

Robust standard errors in parentheses.

*p < .05

** *p* < .01 and

***p < .001.

Amongst the control variables, the coefficients for both employment and joint decision making were significant at <5% level across all specifications. Disability and, likewise, no permanent accommodation, were associated with less positive financial behaviour. By contrast, culture (defined as English-speaking at home individuals) and age (defined as 55–65 = 0, and 65 and above = 1) did not have a significant effect on financial behaviour. The latter may be explained by the low frequency of non-English speaking individuals (5%) and limited age range (see Table 2). Finally, income had a positive and significant effect on financial behaviour across all specifications.

A logistic regression approach following Eqs (2) and (3) was used to examine the relationship between each psychological factor and financial behaviour with maximum PCA weightings. The average marginal effects of coefficients are presented in Table 5. While Specifications (1)-(3) corresponded to the first item of financial behaviour, i.e. *I am very thorough in my approach to financial planning* as the dependent variable, Specifications (4)-(6) corresponded to the second item of financial behaviour, i.e. *I always pay my credit card off each month* as the dependent variable. Both items were considered to check whether the effect of psychological factors such as hope, coping, and mental wellbeing, were heterogenous across different types of the financial behaviour.

**Table 5:** Logit regression estimation (average marginal effect).

Variables	Financial behaviour 1 (*I am very thorough in my approach to financial planning)*			Financial behaviour 2 (*I always pay my credit card off each month)*		
	(1)	(2)	(3)	(4)	(5)	(6)
**Mental Wellbeing**	0.211**			0.167*		
*I have felt cheerful and in good spirits*	(0.076)			(0.137)		
**Hope**		0.118***			0.115	
*Even when others are discouraged*, *I know I can find a way to solve the problem*		(0.030)			(0.081)	
**Cope**			0.155**			-0.115*
*Think about yourself in a less critical*, *harsh or a negative way*			(0.065)			(0.065)
**Gender (women)**	0.100	0.041	0.155	0.115*	0.055	0.056
	(0.99)	(0.011)	(0.095)	(0.078)	(0.051)	(0.035)
**Unemployed**	-0.031	-0.011	-0.156***	-0.601*	-0.558	-0.156
	(0.014)	(0.015)	(0.055)	(0.185)	(0.401)	(0.061)
**Speak English**	0.547	0.041	0.085	0.181*	0.030**	0.018
	(0.471)	(0.011)	(0.055)	(0.071)	(0.011)	(0.011)
**Rent/Mortgage**	0.088	0.011	-0.058**	0.111	0.053	-0.165*
	(0.019)	(0.015)	(0.015)	(0.061)	(0.011)	(0.051)
**Joint decision making**	0.031*	0.151*	-0.017	0.117*	0.081***	-0.068
	(0.014)	(0.055)	(0.015)	(0.058)	(0.010)	(0.051)
**Disability**	-0.441*	-0.015	-0.005	-0.066	-0.011	-0.115*
	(0.107)	(0.010)	(0.001)	(0.057)	(0.011)	(0.051)
**Age group (+65y)**	0.347***	0.064	0.036*	0.318	-0.058	0.158
	(0.113)	(0.051)	(0.015)	(0.163)	(0.011)	(0.081)
**Income**	0.313*	0.450*	0.048**	0.051*	0.050*	0.150
	(0.186)	(0.180)	(0.015)	(0.015)	(0.015)	(0.065)
**N**	1501	1501	1501	1501	1501	1501
**Pseudo R** ^**2**^	0.417	0.531	0.436	0.431	0.474	0.411

Robust standard errors in parentheses.

*p < .05

** *p* < .01 and

*** *p* < .001.

Analyses indicated that when individuals reported feeling calm and relaxed (which was an indicator of increased mental wellbeing) there was a positive effect on both types of financial behaviour (i.e., (i) they are very thorough with their financial planning and (ii) always pay their credit card on time). The coefficients were 0.211 in Specification (1) and 0.167 in Specification (4), which were significant at <1% and <5% levels. Regarding the hope item, when individuals were positive about finding solutions to their problems even when they were discouraged (Hope), they reported more positive financial behaviour 0.118 in Specification (2) and 0.115 in Specification (5). While the coefficient of Hope in Specification (2) was significant at a <0.1% level, the coefficient in Specification (5) was not statistically significant.

Regarding the coping item, individuals who were less critical about themselves (Cope) were more thorough with their financial planning (0.155 at <1% level in Specification (3)), however, a high level of positive coping was shown to negatively impact timely credit card payments (-0.115 at <5% level in Specification (6)). Overall, significant heterogeneity was observed across the effects of psychological factors on financial behaviour.

The control variables typically showed expected effects on financial behaviour as shown in Table 5. Consistent with the OLS results in Table 4, women reported more positive financial behaviour as compared to men in Specification (4), as did those older than 65 compared to 55–65-year-old (Specifications (1) and (3)) and those with a high, as compared to low income, where the coefficient of income was consistently positive and significant across all the specifications, besides Specification (6) (Table 5). In contrast, unemployment and disability were associated with less positive financial behaviour. Renting/mortgaging homes was associated with poorer coping. Finally, English speaking was insignificant across most specifications, apart from Specifications (4) and (5) suggesting that cultural background was a very weak predictor of positive financial behaviour.

### Sensitivity checks

The main findings from the logit regression were checked using an array of sensitivity tests. First, the relationship between psychological factors and financial behaviour were reported to vary according to gender, with men more likely to make risky financial decisions [46] and women to be financially vulnerable post-retirement, particularly after their partner’s death [47]. Hence, the data for women was checked separately and reported in supporting information (S1 Table). The results indicated that for women–mental wellbeing was an important predictor of thorough financial planning approach but not for credit card payments. Conversely, for hope (*Even when others are discouraged*, *I know I can find a way to solve the problem*) the average marginal effect was positive and significant for only Specification (5) i.e., timely payment of credit card every month. By contrast, the coefficient of coping (thinking in a less critical, harsh, or negative way about themselves) did not remain significant for the thorough approach to financial planning in Specification (3) and became negative for timely credit card payments in Specification (6).

Next, the sample was split based on accommodation status, and since renting or having a mortgage could place additional financial and health burdens, this group was considered separately [48]. Further, on the assumption that sharing financial decision-making process would result in higher hope, coping, and mental wellbeing the participants who jointly made financial decisions with their spouses/partner were also considered separately [49]. These results are presented in supporting information, S2 and S3 Tables respectively.

The results in S2 and S3 Tables suggested that mental wellbeing and hope, were positive and significant in all specifications. Also, consistent with Table 5, Coping (*Think about yourself in a kind*, *encouraging and positive way*) was positively associated with systematic financial planning approach (Specification (3)) but negatively associated with on-time credit card payments (Specification (6)) in S1 Table). However, the coefficient lost its significance in Specification (6) of S3 Table when joint decision-making was considered.

In supporting information S4 Table, mental wellbeing was replaced by financial wellbeing, to check whether the average marginal effect on financial behaviour was significantly different for the entire sample. No significant difference was observed compared to baseline findings. However, the magnitude of mental wellbeing in Table 5 (0.211 in Specification (1) and 0.167 in Specification (4)) remained higher than financial wellbeing in S4 Table (0.123 and 0.136).

Finally, endogeneity was controlled for in the analyses and Eqs (2) and (3) were re-estimated using TSLS estimation technique to identify the endogenous variables, i.e., mental wellbeing, hope, and coping. These results are presented in Table 6. The first stage regression results corresponding to Table 6 is provided in the supporting document (S5 Table). Together with the first stage regression results, the instruments passed both the relevancy and exogeneity conditions. Further, Table 6 showed that after controlling for endogeneity, the coefficients of mental wellbeing, hope, and coping were positive and statistically significant. For mental wellbeing, the coefficients were 0.111 in Specification (1) and 0.114 in Specification (4), which were highly significant at <0.1% level. Similarly, for the hope item, the coefficients were 0.073, and 0.068 in Specifications (2) and (5) respectively, which are all significant at <1% level. With reference to the coping items, after controlling for endogeneity, the coefficient of Coping (*Think about yourself in a kind*, *encouraging and positive way*) in Specification (3) turned out to be positive, though not significant in Table 6, which was previously negative and significant in Table 5. Hence, it can be concluded that the estimate for Coping in Table 5 had endogeneity bias, which got rectified in Table 6. Overall, after controlling for endogeneity the results were consistent with our baseline findings in Table 5, with positive mental wellbeing and hope as strong predictors of positive financial behaviour.

**Table 6:** Two-stage least square estimation.

Variables	Financial behaviour 1 *(I am very thorough in my approach to financial planning)*			Financial behaviour 2 *(I always pay my credit card off each month*		
	(1)	(2)	(3)	(4)	(5)	(6)
**Mental Wellbeing**	0.111***			0.114***		
**Item 4:** *I have woken up feeling fresh and rested*	(0.018)			(0.016)		
**Hope**		0.073**			0.068**	
**Item 5:** *I do not feel tired most of the time*		(0.018)			(0.014)	
**Cope**			0.086			0.050
**Item 4:** *Pray*			(0.045)			(0.018)
**Women**	0.081	0.044	0.037	0.068	0.054	0.041
	(0.078)	(0.078)	(0.077)	(0.061)	(0.061)	(0.061)
**Unemployed**	-0.037	-0.050	-0.051	-0.106	-0.088	-0.087*
	(0.047)	(0.047)	(0.047)	(0.077)	(0.087)	(0.047)
**Speak English**	0.414	0.467	0.461	0.384	0.414	0.440
	(0.331)	(0.334)	(0.334)	(0.161)	(0.164)	(0.164)
**Rent/Mortgage**	0.057**	0.063**	0.064**	0.087**	0.081**	0.081**
	(0.018)	(0.018)	(0.018)	(0.014)	(0.015)	(0.014)
**Joint decision making**	0.005	0.004	0.004	0.005	0.008	0.008
	(0.015)	(0.015)	(0.015)	(0.011)	(0.011)	(0.011)
**Disability**	-0.073	-0.141	-0.158	-0.113	-0.143*	-0.157*
	(0.088)	(0.088)	(0.086)	(0.068)	(0.070)	(0.068)
**Age group (+65y)**	0.188*	0.177*	0.177*	0.034	0.058	0.063
	(0.080)	(0.081)	(0.081)	(0.064)	(0.064)	(0.064)
**Income**	0.034*	0.034*	0.034*	0.037*	0.078**	0.068**
	(0.017)	(0.017)	(0.017)	(0.013)	(0.014)	(0.014)
**N**	1501	1501	1501	1501	1501	1501
**Pseudo R** ^**2**^	0.614	0.630	0.731	0.678	0.755	0.776
**Cragg-Donald Wald F-statistic test**	106.23	96.15	81.37	102.36	100.20	67.21

Note: Robust standard errors in parentheses.

*p < .05

** *p* < .01 and

*** *p* < .001.

## Discussion

The main findings of the present study were twofold. First, baseline results revealed that higher scores on all the psychological factors were associated with more positive financial behaviour. Thus, confirming our main hypothesis that at times of crises, such as the COVID-19 pandemic, psychological factors such as hope, positive coping, and positive mental wellbeing are associated with positive financial behaviour. Second, sensitivity analyses revealed that hope and mental wellbeing, compared to positive coping were more strongly associated with positive financial behaviours.

This study examined the financial behaviour of older Australians during the early stages of the COVID-19 pandemic and how it was affected by psychological factors. It contributes to the literature in several ways. The study used three different variables, i.e., hope, coping, and mental wellbeing, in one setting to examine how they affect financial behaviour. Studies have investigated these variables independently in multiple settings [50, 51], but none have used these together to examine how they affect financial behaviour in older adults. Further, the study used a unique individual level dataset that involved older demographic responses to psychological wellbeing and financial decision-making in a time of crisis. Although several studies have investigated psychological factors and financial decisions of older people in non-crisis settings [52–54], to the best of our knowledge this study was the first to use a crisis setting—the COVID-19 pandemic.

Analysis of the control variables on socio-economic and demographic characteristics revealed that older people who were employed and those who made joint decisions with their partners (i.e., engaged in financial socialisation) were more likely to report positive financial behaviour. By contrast, cultural background, rental status, and income were weak predictors of positive financial behaviour. Employment and financial socialisation are known predictors of positive financial wellbeing [6, 8]. Consistent with previous findings, women were more likely to report positive financial behaviours (see S1 Table) [46].

Sensitivity analysis revealed that for older people who have financial burdens (e.g., renting and mortgages) and who jointly make financial decisions with their partners, in both instances, positive mental wellbeing and hope were significant predictors for both financial behaviours. By contrast the findings for positive coping were mixed. In older people who were renting/mortgage, positive coping was a significant and positive predictor of thorough financial planning but a negative and significant predictor of timely credit card payment. In older people with a high level of financial socialisation positive coping was a significant predictor of thorough financial planning but a non-significant predictor of timely credit card payment. This may be because those coping strategies are effective for good financial behaviours (see OLS results), but insufficient to support people who are already facing financial burdens. It is possible that positive mental wellbeing and hope, but not positive coping may help individuals better emerge out of a crisis (such as not accumulating additional financial burden or debt).

To check whether the average marginal effect on financial behaviour was significant, mental wellbeing was replaced in the analyses by financial wellbeing as an additional robustness check. This revealed that there was a strong relationship between financial wellbeing and mental wellbeing and that financial wellbeing was a significant predictor of financial behaviour. These relationships are consistent with previous findings [55, 56]. Additional analyses were also undertaken to test whether a reverse causality bias was evident (i.e., if more frequent positive financial behaviour result in greater financial wellbeing, leading individuals to become more hopeful and better able to cope with financial distress). After controlling for endogeneity, a reverse causality bias was not evident with positive coping remaining insignificant but both positive mental wellbeing and hope remaining significant predictors of positive financial behaviours.

This study has several limitations. First, the survey questionnaire was conducted over the telephone which may have biased participant selection [57]. To minimise the non-response bias in the present study, two more calls were made at different times during the day to participants who did not answer a call on the first attempt. Second, stratification of the sample was limited to sex, retirement status, age (55-65y vs 66+y), region (metropolitan/regional) and location (all major Australian capital cities). As such, groups vulnerable to financial distress such as those with disability and Indigenous Australians were not purposively sampled. It is noted that proportionately more of the sample were from middle and high SES groups, which may have contributed to higher financial wellbeing scores. A final limitation is that this was a cross-sectional study with the problem attendant to such a research design. There is a need for longitudinal studies to predict the long-term impact in older people of the COVID-19 pandemic on psychological wellbeing and financial behaviour over multiple waves and into the post-COVID-19 period. Finally, it is noted that the interview was scripted and administered by trained interviewers, sampled across a broad socioeconomic range, employed a survey method likely to recruit older participants (as compared to online), and overcame the COVID-19 restrictions on face-to-face interviews present at the time [58].

## Conclusions

In conclusion, the study found that hope and positive mental wellbeing and, to a lesser extent, coping, were predictors of positive financial behaviour. In addition, it was found that two items, one each assessing hope and positive mental wellbeing, could be aggregated into an index that predicted financial behaviour raising the possibility of a simple index for use in future studies involving older people. The COVID-19 pandemic has presented older people with psychological and financial challenges. The present findings suggest that the two are interlinked and raise the possibility that promoting psychological wellbeing may facilitate positive financial behaviour in a vulnerable group at a time of crisis.

## Supporting information

S1 Table Sensitivity Test 1. Logit Regression Estimation (Average marginal effect for women only).
(DOCX)

S2 Table Sensitivity Test 2. Logit Regression Estimation (Average marginal effect–Rent/Mortgage Data Only).
(DOCX)

S3 Table Sensitivity Test 3. Logit Regression Estimation (Average marginal effect–Joint Decision-Making Data Only).
(DOCX)

S4 Table Sensitivity Test 4. Logit Regression Estimation (Average marginal effect—Financial Wellbeing).
(DOCX)

S5 Table First-stage least square estimation. (DOCX)
