# Supplementary material for: The effect of psychological factors on financial behaviour among older Australians: Evidence from the early stages of COVID-19 pandemic
Source: PLoS One. 2023 Jun 8;18(6):e0286733. doi: 10.1371/journal.pone.0286733 (PMC10249876; doi:10.1371/journal.pone.0286733)
Supplement: S1 Table — Logit Regression Estimation (Average marginal effect for women only). (DOCX) [file pone.0286733.s001.docx]

**S1 Table. Sensitivity Test 1. Logit Regression Estimation** (Average marginal effect for women only).

| ***Variables*** | ***Financial behaviour 1*** *(I am very thorough in my approach to financial planning)* | | | ***Financial behaviour 2*** *(I* *always pay my credit card off each month)* | | | |
| --- | --- | --- | --- | --- | --- | --- | --- |
|  | ***(1)*** | ***(2)*** | ***(3)*** | ***(4)*** | ***(5)*** | ***(6)*** |  |
| **Mental Wellbeing** | 0.018** |  |  | 0.018 |  |  |  |
| *I have felt cheerful and in good spirits* | (0.007) |  |  | (0.017) |  |  |  |
| **Hope** |  | 0.008 |  |  | 0.035* |  |  |
| *Even when others are discouraged, I know I can find a way to solve the problem* |  | (0.005) |  |  | (0.010) |  |  |
| **Cope** |  |  | 0.055 |  |  | -0.005 |  |
| *Think about yourself in a less critical, harsh or a negative way* |  |  | (0.035) |  |  | (0.065) |  |
|  |  |  |  |  |  |  |  |
| **Unemployed** | -0.010 | -0.000 | -0.056** | -0.110* | -0.558 | -0.056 |  |
|  | (0.004) | (0.005) | (0.055) | (0.085) | (0.400) | (0.030) |  |
| **Speak English** | 0.007 | 0.040 | 0.085 | 0.080* | 0.010* | 0.008 |  |
|  | (0.070) | (0.000) | (0.055) | (0.070) | (0.000) | (0.006) |  |
| **Rent/Mortgage** | 0.088 | 0.000 | -0.058** | 0.080 | 0.051 | -0.065* |  |
|  | (0.041) | (0.005) | (0.005) | (0.060) | (0.015) | (0.010) |  |
| **Joint decision making** | 0.010* | 0.050* | -0.007 | 0.087* | 0.080*** | -0.068 |  |
|  | (0.004) | (0.055) | (0.005 | (0.058) | (0.000) | (0.050) |  |
| **Disability** | -0.016 | -0.005 | -0.005 | -0.066 | -0.037 | -0.005 |  |
|  | (0.007) | (0.004) | (0.000) | (0.057) | (0.015) | (0.003) |  |
| **Age group (+65y)** | 0.147*** | 0.064 | 0.016* | 0.108 | -0.056 | 0.058 |  |
|  | (0.001) | (0.050) | (0.005) | (0.061) | (0.041) | (0.040) |  |
| **Income** | 0.101* | 0.150* | 0.006 | 0.050* | 0.050* | 0.050 |  |
|  | (0.086) | (0.080) | (0.005) | (0.011) | (0.014) | (0.041) |  |
| **N** | 751 | 751 | 751 | 751 | 751 | 751 |  |
| **Pseudo R^2^** | 0.587 | 0.620 | 0.656 | 0.650 | 0.696 | 0.653 |  |

*Note*: Robust standard errors in parentheses.**p* < .05, ** *p* < .01 and *** *p* < .001.
