# Supplementary material for: The effect of psychological factors on financial behaviour among older Australians: Evidence from the early stages of COVID-19 pandemic
Source: PLoS One. 2023 Jun 8;18(6):e0286733. doi: 10.1371/journal.pone.0286733 (PMC10249876; doi:10.1371/journal.pone.0286733)
Supplement: S2 Table — Logit Regression Estimation (Average marginal effect–Rent/Mortgage Data Only). (DOCX) [file pone.0286733.s002.docx]

**S2 Table. Sensitivity Test 2. Logit Regression Estimation** (Average marginal effect – Rent/Mortgage Data Only).

| **Variables** | **Financial behaviour 1** *(I am very thorough in my approach to financial planning)* | | | **Financial behaviour 2** *(I always pay my credit card off each month)* | | |
| --- | --- | --- | --- | --- | --- | --- |
|  | **(1)** | **(2)** | **(3)** | **(4)** | **(5)** | **(6)** |
| **Mental Wellbeing** | 0.035** |  |  | 0.134** |  |  |
| *I have felt cheerful and in good spirits* | (0.005) |  |  | (0.011) |  |  |
| **Hope** |  | 0.081** |  |  | 0.084** |  |
| *Even when others are discouraged, I know I can find a way to solve the problem* |  | (0.011) |  |  | (0.017) |  |
| **Cope** |  |  | 0.056** |  |  | -0.131** |
| *Think about yourself in a less critical, harsh or a negative way* |  |  | (0.010) |  |  | (0.010) |
| **Women** | 0.001 | 0.048 | 0.053 | 0.011 | -0.013 | 0.005 |
|  | (0.014) | (0.047) | (0.048) | (0.037) | (0.038) | (0.038) |
| **Unemployed** | -0.001 | -0.048 | -0.053 | -0.011 | -0.013 | -0.005 |
|  | (0.014) | (0.047) | (0.048) | (0.037) | (0.038) | (0.038) |
| **Speak English** | 0.000 | 0.014 | 0.011 | 0.055 | 0.054 | 0.053 |
|  | (0.008) | (0.015) | (0.016) | (0.041) | (0.051) | (0.031) |
| **Joint decision making** | 0.031** | 0.058* | 0.061* | 0.087** | 0.081*** | 0.081*** |
|  | (0.008) | (0.018) | (0.018) | (0.013) | (0.014) | (0.013) |
| **Disability** | -0.001 | -0.018 | 0.014 | 0.014 | -0.013 | -0.013 |
|  | (0.010) | (0.036) | (0.036) | (0.018) | (0.018) | (0.018) |
| **Age group (65+)** | 0.016 | 0.111* | 0.114* | 0.017 | 0.030 | 0.045 |
|  | (0.015) | (0.051) | (0.051) | (0.041) | (0.043) | (0.041) |
| **Income** | 0.007 | 0.086 | 0.088 | 0.007 | 0.088 | 0.074 |
|  | (0.015) | (0.050) | (0.050) | (0.004) | (0.041) | (0.041) |
| **N** | 471 | 471 | 471 | 471 | 471 | 471 |
| **Pseudo R^2^** | 0.611 | 0.613 | 0.531 | 0.646 | 0.618 | 0.611 |

*Note*: Robust standard errors in parentheses. **p* < .05, ** *p* < .01 and *** *p* < .001.
