# Supplementary material for: The effect of psychological factors on financial behaviour among older Australians: Evidence from the early stages of COVID-19 pandemic
Source: PLoS One. 2023 Jun 8;18(6):e0286733. doi: 10.1371/journal.pone.0286733 (PMC10249876; doi:10.1371/journal.pone.0286733)
Supplement: S3 Table — Logit Regression Estimation (Average marginal effect–Joint Decision-Making Data Only). (DOCX) [file pone.0286733.s003.docx]

**S3 Table. Sensitivity Test 3. Logit Regression Estimation** (Average marginal effect – Joint Decision-Making Data Only).

| **Variables** | **Financial behaviour 1** *I am very thorough in my approach to financial planning* | | | **Financial behaviour 2** *I always pay my credit card off each month* | | |
| --- | --- | --- | --- | --- | --- | --- |
|  | **(1)** | **(2)** | **(3)** | **(4)** | **(5)** | **(6)** |
| **Mental Wellbeing** | 0.118** |  |  | 0.167** |  |  |
| *I have felt cheerful and in good spirits* | (0.066) |  |  | (0.053) |  |  |
| **Hope** |  | 0.146** |  |  | 0.148*** |  |
| *Even when others are discouraged, I know I can find a way to solve the problem* |  | (0.040) |  |  | (0.033) |  |
| **Cope** |  |  | 0.017* |  |  | 0.037 |
| *Think about yourself in a less critical, harsh or a negative way* |  |  | (0.007) |  |  | (0.013) |
| **Women** | 0.004 | 0.018 | 0.036 | 0.018 | 0.001 | 0.008 |
|  | (0.051) | (0.047) | (0.048) | (0.041) | (0.038) | (0.040) |
| **Unemployed** | -0.018 | -0.010 | -0.011 | -0.061** | -0.068*** | -0.060*** |
|  | (0.018) | (0.016) | (0.016) | (0.013) | (0.011) | (0.011) |
| **Speak English** | 0.381 | 0.168 | 0.148 | 0.013 | 0.163 | 0.186 |
|  | (0.115) | (0.187) | (0.188) | (0.173) | (0.153) | (0.155) |
| **Rent/Mortgage** | 0.051 | 0.067* | 0.070* | -0.081 | 0.057 | -0.100 |
|  | (0.031) | (0.018) | (0.018) | (0.016) | (0.014) | (0.104) |
| **Disability** | -0.088 | -0.111* | -0.071 | -0.016 | -0.051 | -0.055 |
|  | (0.058) | (0.051) | (0.051) | (0.047) | (0.041) | (0.043) |
| **Age group (65+)** | 0.101* | 0.078 | 0.081 | 0.081* | 0.063 | 0.085 |
|  | (0.055) | (0.050) | (0.050) | (0.044) | (0.041) | (0.041) |
| **Income** | 0.073 | 0.040 | 0.081* | 0.040 | 0.073* | 0.061* |
|  | (0.041) | (0.038) | (0.038) | (0.033) | (0.031) | (0.031) |
| **N** | 858 | 858 | 858 | 858 | 858 | 858 |
| **Pseudo R - sq** | 0.517 | 0.550 | 0.561 | 0.588 | 0.575 | 0.476 |

*Note*: Robust standard errors in parentheses. **p* < .05, ** *p* < .01 and *** *p* < .001
