# Supplementary material for: The effect of psychological factors on financial behaviour among older Australians: Evidence from the early stages of COVID-19 pandemic
Source: PLoS One. 2023 Jun 8;18(6):e0286733. doi: 10.1371/journal.pone.0286733 (PMC10249876; doi:10.1371/journal.pone.0286733)
Supplement: S4 Table — Logit Regression Estimation (Average marginal effect—Financial Wellbeing). (DOCX) [file pone.0286733.s004.docx]

**S4 Table.**  **Sensitivity Test 4.** **Logit Regression Estimation** (Average marginal effect - Financial Wellbeing).

| **Variables** | **Financial behaviour 1**  *(I am very thorough in my approach to financial planning)* | **Financial behaviour 2** (*I always pay my credit card off each month)* |
| --- | --- | --- |
| **Financial Wellbeing**  *I am satisfied with my financial situation* | 0.113**  (0.045) | 0.136**  (0.0715) |
| **Gender (women)** | 0.015* | 0.051* |
|  | (0.007) | (0.011) |
| **Unemployed** | 0.015** | 0.016** |
|  | (0.013) | (0.011) |
| **Speak English** | 0.011 | 0.058 |
|  | -0.051 | (0.051) |
| **Rent/Mortgage** | -0.031* | -0.071** |
|  | (0.011) | (0.011) |
| **Joint decision making** | 0.018** | 0.018** |
|  | (0.001) | (0.011) |
| **Disability** | -0.011* | -0.018** |
|  | (0.008)1 | (0.011) |
| **Age group (+65y)** | 0.003 | 0.005 |
|  | (0.006) | (0.004) |
| **Income** | 0.016*** | 0.011*** |
|  | (0.001) | (0.003) |
| R^2^ | 0.611 | 0.615 |
| N | 1501 | 1501 |

*Note:* Robust standard errors in parentheses. **p* < .05, ** *p* < .01 and *** *p* < .001.
