# Supplementary material for: The effect of psychological factors on financial behaviour among older Australians: Evidence from the early stages of COVID-19 pandemic
Source: PLoS One. 2023 Jun 8;18(6):e0286733. doi: 10.1371/journal.pone.0286733 (PMC10249876; doi:10.1371/journal.pone.0286733)
Supplement: S5 Table — (DOCX) [file pone.0286733.s005.docx]

**S5 Table. First -Stage Least Square Estimation.**

| **Variables** | **Mental Wellbeing** | **Hope** | **Cope** |
| --- | --- | --- | --- |
| **IV1** (Mental wellbeing): *I have woken up feeling fresh and rested* | 0.151*** |  |  |
|  | (0.035) |  |  |
| **IV2** (Hope): *I do not feel tired most of the time* |  | 0.107*** |  |
|  |  | (0.041) |  |
| **IV3** (Cope): *Pray* |  |  | 0.131*** |
|  |  |  | (0.038) |
| **Women** | 0.188*** | 0.117 | 0.178*** |
|  | (0.056) | (0.080) | (0.068) |
| **Unemployed** | -0.074* | -0.040 | -0.048 |
|  | (0.034) | (0.048) | (0.041) |
| **Speak English** | 0.133 | 0.548 | 0.078 |
|  | (0.143) | (0.345) | (0.181) |
| **Rent/Mortgage** | 0.065*** | 0.048* | 0.055*** |
|  | (0.013) | (0.018) | (0.016) |
| **Joint decision making** | 0.013 | 0.011 | 0.011 |
|  | (0.011) | (0.016) | (0.013) |
| **Disability** | -0.318*** | -0.061 | -0.617*** |
|  | (0.063) | (0.088) | (0.075) |
| **Age group (65+)** | 0.145* | 0.161 | 0.146* |
|  | (0.058) | (0.083) | (0.071) |
| **Income** | 0.013 | 0.045* | 0.011 |
|  | (0.011) | (0.018) | (0.015) |
| **N** | 1501 | 1501 | 1501 |
| **R^2^** | 0.771 | 0.617 | 0.601 |

*Note*: Robust standard errors in parentheses. IV = independent variable. **p* < .05, ** *p* < .01 and *** *p* < .001.
